# Psychiatric Comorbidities in Autistic Adolescents Without Intellectual Impairment: A Focus on Parent- and Self-Reported Psychopathological Assessment

**DOI:** 10.3390/brainsci15020187

**Published:** 2025-02-13

**Authors:** Romina Cagiano, Alice Mancini, Marta Berni, Federica Maccarrone, Benedetta Arena, Angela Cosenza, Chiara Pecini, Roberta Igliozzi, Sara Calderoni, Raffaella Tancredi

**Affiliations:** 1 Istituto di Ricovero e Cura a Carattere Scientifico (IRCCS) Stella Maris Foundation, Calambrone, 56128 Pisa, Italy; romina.cagiano@fsm.unipi.it (R.C.); alice.mancini@fsm.unipi.it (A.M.); marta.berni1@unifi.it (M.B.); federica.maccarrone@fsm.unipi.it (F.M.); benedetta.arena@fsm.unipi.it (B.A.); angela.cosenza@fsm.unipi.it (A.C.); roberta.igliozzi@fsm.unipi.it (R.I.); sara.calderoni@unipi.it (S.C.); 2 University of Florence, 50121 Florence, Italy; 3 Department of Clinical and Experimental Medicine, University of Pisa, 56126 Pisa, Italy; 4 Department of Education, Languages, Intercultures, Literatures and Psychology (FORLILPSI), University of Florence, 50121 Florence, Italy; chiara.pecini@unifi.it

**Keywords:** autism, comorbidities, psychopathological assessment

## Abstract

**Background:** Co-occurring conditions and psychiatric comorbidities are more frequently observed in autistic individuals than in typically developing populations. **Objective:** The present study aimed to investigate the agreement of parent- and self-reported psychopathological assessment using the Child Behavior Checklist (CBCL/6-18) and the Youth Self Report (YSR/11-18), respectively, in autistic adolescents without intellectual impairment. **Methods:** 54 autistic adolescents without intellectual impairment (11–18 years; M = 14.73; SD = 2.28) were assessed with a psychiatric and psychological evaluation conducted by expert clinicians also using self- and parent-reported scales and semi-structured interviews (K-SADS PL, CDI, MASC) including CBCL/6-18 and YSR/11-18. **Results:** According to clinical judgment, over 90% of participants had at least a comorbidity: anxiety (68.5%) and mood disorder (57.4%) were the most frequent. The results indicate significant discrepancies between parent- and self-reports across the three summary scales, which assess emotional and behavioral problems, as well as their combined presentation, often observed in youth with ASD. Specifically, differences were found in Internalizing (*p* < 0.001), Externalizing (*p* = 0.013), and Total Problems (*p* < 0.001) scales. **Conclusions:** The findings show the lack of agreement in parent- and self-reported scales in our sample. These results suggest the need for a cross- and multi-informant approach to support clinical judgment and understand psychopathological comorbidities of autistic adolescents without intellectual impairment.

## 1. Introduction

Autism spectrum disorder (ASD) is a neurodevelopmental disorder characterized by early-onset differences in social communication and reciprocal interaction, as well as restricted interests and repetitive behaviors [1]. Its global prevalence is estimated to be approximately 1% [2]. Autistic individuals are more likely to experience co-occurring conditions compared to the typically developing population. These conditions often include other neurodevelopmental differences (e.g., intellectual disability), as well as medical, psychological, and behavioral challenges [3].

Systematic reviews and meta-analyses have examined the prevalence of psychiatric comorbidities among autistic individuals, revealing a wide range of estimates. Research indicates that autistic people are at significantly higher risk of developing psychiatric conditions compared to the general population [4,5]. Studies suggest that 70% of autistic individuals present with at least one comorbid psychiatric condition, while nearly 40% may have two or more psychiatric diagnoses [6].

Among these, Attention Deficit/Hyperactivity Disorder (ADHD) is the most prevalent, followed by anxiety disorders, sleep–wake disorders, disruptive, impulse-control, and conduct disorders, depressive disorders, obsessive-compulsive disorder, bipolar disorders, and schizophrenia spectrum disorders [3,4,5,7,8]. Notably, studies focusing on autistic adolescents without intellectual disabilities have found that individuals with heightened perceptual abilities are particularly likely to have at least one psychiatric comorbidity [9,10]. Several studies have highlighted the significant impact of psychiatric conditions on both short- and long-term outcomes for autistic people. These include the exacerbation of autistic traits and behavioral challenges, leading to greater difficulties in daily life [11]; negative effects on adaptive and social functioning [10,12]; poorer long-term outcomes [13]; and an increased risk of mortality [14].

The substantial heterogeneity of psychiatric comorbidities in autism, as described in the literature, can be attributed to multiple factors, including study design, sample characteristics, differences in the phenotypic presentation of these conditions in autism, the methodologies used to assess psychiatric disorders, and the limited availability of assessment tools specifically designed for autistic people [15]. Individual factors contributing to variability include age [6], gender [16], cognitive abilities [17], and the severity of autistic traits [18,19]. Indeed, core autistic traits can sometimes mask co-occurring psychiatric symptoms and vice versa [20]. Additionally, the phenomenon of camouflaging, understood as a coping strategy used to mask autistic traits in social situations, has been associated with the occurrence of internalizing symptoms in autistic children and adolescents [21].

Age is a crucial factor in understanding the developmental trajectories and outcomes of psychiatric conditions in autism. Several studies indicate that both the prevalence and manifestation of psychiatric comorbidities increase with age [4,22,23]. During adolescence, the most frequently observed psychiatric conditions are mood and anxiety disorders, followed by ADHD [4,11].

The assessment of psychiatric comorbidities has gained increased attention following the introduction of the DSM-5, which removed previous restrictions on additional diagnoses alongside ASD [24]. Diagnosing co-occurring conditions remains inherently challenging, as autism itself is a complex neurodevelopmental condition, and psychiatric comorbidities become even more difficult to identify in autistic individuals without intellectual disabilities or language impairments due to their diverse clinical presentations [25]. In clinical practice, psychiatric comorbidity assessment in autism should involve direct observation, semi-structured clinical interviews, and questionnaires administered to both autistic individuals and their caregivers. However, the lack of appropriate assessment tools, atypical symptom presentations, and symptom overlap can complicate the evaluation of mental health conditions in autism, potentially preventing individuals from receiving necessary support [26,27,28,29,30].

There is ongoing debate regarding interoceptive accuracy in autistic individuals, with mixed findings. While some studies suggest reduced interoceptive accuracy in autism [31], others report no significant differences compared to non-autistic populations [32]. Research on co-occurring emotional and behavioral challenges in autistic children and adolescents frequently relies on reports from parents or teachers [33]. However, gathering information from multiple informants (e.g., parents, teachers, and the adolescents themselves) is considered the gold standard for diagnosing mental disorders and improving treatment outcomes [34,35]. Inter-informant agreement has been extensively studied in non-autistic youth, particularly in comparisons between parents, teachers, and children, as well as between both parents [36,37].

Stratis (2015) conducted a meta-analysis on multiple-informant agreement in autistic populations and those with intellectual disabilities (ID) [38]. The findings revealed that, similar to non-autistic populations, the average level of agreement between parents and their children in self-reports is lower than that observed between informants of the same type (e.g., two parents). This discrepancy is likely influenced by factors such as the diagnosis, age, and IQ of the autistic child or adolescent. Notably, among the studies analyzed, only a small proportion specifically examined agreement between parents and their autistic children or adolescents.

The literature presents conflicting findings regarding the level of agreement between autistic adolescents’ self-reports and parental reports on behavioral and emotional challenges. Empirical evidence suggests moderate to strong agreement between autistic adolescents and their parents when reporting behavioral and emotional challenges using the ASEBA parent and adolescent forms [32]. In one study, 45 autistic adolescents with cognitive abilities ranging from below to above average demonstrated shared insight and understanding with their parents regarding their emotional experiences and behaviors.

However, some studies have reported lower agreement among different informants. One study examined autistic adolescents aged 11–17 years, along with their parents and teachers, using the Youth Self-Report (YSR), the parent-rated Child Behavior Checklist (CBCL), and the Teacher Report Form (TRF). The findings revealed weak to moderate correlations between self-reports and parent reports on social and externalizing problems, with even lower agreement regarding anxiety and depressive symptoms [39].

Similarly, another study examined 50 adolescent–parent dyads (n = 26 autistic, n = 24 non-autistic; ages 12–16 years; IQ > 80), where both groups completed parallel versions of an anxiety scale. Autistic adolescents reported higher levels of anxiety than both their non-autistic peers and their own parents [20]. In contrast, Pisula (2017), who included parents of adolescent participants and employed the CBCL (4–18) and YSR (11–18), found that autistic adolescents perceived their challenges as less severe compared to their parents’ assessments [40].

In the Italian context, few studies have examined autistic adolescents without intellectual and language impairments, aiming to describe psychiatric comorbidities while integrating both parental and self-reported perspectives.

To our knowledge, this is the first study to investigate parent–adolescent agreement on psychopathological assessments in a sample of Italian autistic adolescents without intellectual disabilities, using the Child Behavior Checklist (CBCL/6–18) completed by parents and the Youth Self-Report (YSR/11–18) completed by adolescents [41].

## 2. Materials and Methods

### 2.1. Sample

This study retrospectively collected aggregated data from assessments of autistic adolescents conducted at an Italian tertiary care university hospital between November 2015 and August 2024. These assessments were part of a specialized diagnostic pathway designed for autistic children and adolescents without cognitive or speech impairments, with the aim of diagnosing and monitoring their developmental trajectories.

Each participant underwent a comprehensive evaluation conducted by a team of neuropsychiatrists and psychologists with expertise in autism assessment and developmental psychopathology. The standardized assessment protocol included a neuropsychiatric examination, an evaluation of cognitive and adaptive functioning, a specific assessment of autistic traits, and a comprehensive psychopathological evaluation. The psychopathological assessment was based on direct observation, semi-structured clinical interviews, and questionnaires administered separately to parents and adolescents. Among the tools used were the Kiddie Schedule for Affective Disorders and Schizophrenia for School-Age Children—Present and Lifetime Version (K-SADS-PL) [42], the Multidimensional Anxiety Scale for Children, 2nd Edition (MASC-2) [43,44], the Children’s Depression Inventory, 2nd Edition (CDI-2) [45], as well as the Child Behavior Checklist (CBCL/6–18) [41] and the Youth Self-Report (YSR/11–18) [41].

Participants were selected from a larger cohort based on specific inclusion criteria. Eligible individuals were between 11 and 18 years old and had received a clinical diagnosis of ASD according to DSM-5 criteria, confirmed through gold-standard instruments such as the Autism Diagnostic Observation Schedule, 2nd Edition (ADOS-2) [46], and the Autism Diagnostic Interview-Revised (ADI-R) [47]. Additionally, they had a Full-Scale Intelligence Quotient (IQ) of 85 or above, assessed through individualized, standardized intelligence testing, and did not present an expressive or receptive language disorder.

The sample size was verified according to the existing literature [37,38], and a power analysis was conducted using G*Power 3(power level = 0.90, effect size (dz) = 0.45, α = 0.05, estimate sample size = 57; [48]). The initial sample included 57 adolescents; however, 3 participants were unable to complete enough YSR items to yield valid results. To assess the potential impact of this reduction, a post hoc power analysis was conducted, confirming that the study maintained adequate power to detect the expected effects (power level = 0.89, effect size (dz) = 0.45, α = 0.05, estimate sample size = 54; [48]).

The final sample comprised 54 adolescents (44 males and 10 females), with a chronological age range of 11.11 to 18.86 years (M = 14.73, SD = 2.28).

All participants, along with their parents or legal guardians when applicable, provided written informed consent for clinical care and data collection for research purposes.

### 2.2. Measures

For this study, we analyzed data exclusively from the Achenbach System of Empirically Based Assessment (ASEBA) questionnaires, which were completed by both adolescents and one of their parents. These tools are among the most widely used measures for assessing behavioral and emotional symptoms in international epidemiological research and are commonly applied in psychopathological screening for autistic children and adolescents. Specifically, adolescents completed the Youth Self-Report (YSR/11–18) [41], while parents completed the Child Behavior Checklist (CBCL/6–18) [41].

#### 2.2.1. Child Behavior Checklist

The Child Behavior Checklist (CBCL) is a widely used caregiver-report measure designed to assess emotional and behavioral difficulties in children and adolescents. As part of the Achenbach System of Empirically Based Assessment (ASEBA), it has been shown to be effective in identifying these challenges in autistic children and adolescents, despite originally being developed for the general pediatric population.

In this study, we used the CBCL/6–18, which is designed for individuals aged 6 to 18 years. This instrument consists of a 113-item parent-report questionnaire rated on a three-point scale, providing scores across broad domains—internalizing and externalizing difficulties—as well as more specific symptom categories. The scoring system accounts for age and sex differences, ensuring comparability across diverse populations.

The CBCL includes two main sets of subscales. The Syndromic Scales comprise eight categories: Anxious/Depressed, Withdrawn/Depressed, Somatic Complaints, Social Problems, Thought Problems, Attention Problems, Rule-Breaking Behavior, and Aggressive Behavior. Meanwhile, the DSM-Oriented Scales include six subscales: Affective Problems, Anxiety Problems, Somatic Problems, Attention Deficit/Hyperactivity Problems, Oppositional Defiant Problems, and Conduct Problems. In addition, three newer scales introduced in 2007—Sluggish Cognitive Tempo, Obsessive-Compulsive Problems, and Post-Traumatic Stress Problems—offer further insight into specific behavioral and emotional patterns. The overall Internalizing Problems and Externalizing Problems scores are derived from a combination of these subscales, and a Total Problem Score is calculated by summing all problem items. For the purposes of this study, open-ended responses and the Sluggish Cognitive Tempo subscale were not analyzed.

The CBCL/6–18 can be scored manually or through the Computerized Assessment Data Manager (ADM) software 9.1 Version, which aggregates individual item responses to generate Syndromic Scales, DSM-Oriented Scales, Summary Scales, and the Stress Problems Scale. Each scale is reported in percentile scores and T-scores, with clinical cutoffs distinguishing different levels of concern. A T-score above 69 indicates a clinically significant range for the Syndromic Scales, DSM-Oriented Scales, and Stress Problems Scale, while a T-score above 63 denotes clinical significance for the Summary Scales. The borderline range is defined by a T-score between 65 and 69 for the Syndromic Scales, DSM-Oriented Scales, and Stress Problems Scale and between 60 and 63 for the Summary Scales.

For this study, we used the Italian adaptation of the CBCL/6–18, developed by Frigerio and colleagues (2001) [49].

#### 2.2.2. Youth Self-Report

The Youth Self-Report (YSR/11–18) is a self-assessment questionnaire designed for adolescents aged 11 to 18 to evaluate their behavioral and emotional difficulties. Developed as the self-report counterpart to the Child Behavior Checklist (CBCL), it consists of 112 first-person items that assess a wide range of psychological and behavioral concerns. The YSR/11–18 provides scores across multiple domains, including broad categories such as internalizing and externalizing difficulties, as well as more specific symptom subgroups.

The structure of the YSR/11–18 closely aligns with that of the CBCL/6–18, incorporating similar scales while also introducing additional subscales in 2007, such as Obsessive-Compulsive Problems, Post-Traumatic Stress Problems, and Positive Qualities. In this study, only the scored scales were analyzed, while open-ended responses and the Positive Qualities subscale were excluded. The YSR/11–18 can be scored manually or through computerized software, producing percentile ranks and T-scores. A T-score above 69 is considered within the clinical range for most scales, while scores between 65 and 69 indicate borderline clinical significance.

For this study, we used the Italian adaptation of the YSR/11–18, translated and validated by Frigerio and colleagues in 2001 [49]. Both the CBCL and YSR have demonstrated strong psychometric properties in research involving autistic adolescents [50,51].

### 2.3. Data Analysis

All statistical analyses were performed using the Statistical Package for the Social Sciences (SPSS, version 29.0.2.0). To assess whether the data followed a normal distribution, we performed a Kolmogorov–Smirnov test, which was deemed appropriate given the sample size (n > 50). Descriptive and frequency analyses were carried out on the standardized scores, and missing data were not interpolated. To investigate potential age-related differences, we conducted Spearman’s rank correlation analyses to assess the relationship between age and the key variables of interest.

Wilcoxon Signed-Rank Tests were conducted to compare test scores from the Child Behavior Checklist and the Youth Self-Report across the Total Problems, Internalizing Problems, and Externalizing Problems subscales. Additionally, the effect size (r) was calculated. According to Cohen’s guidelines for r, an effect size of 0.5 is considered large, 0.3 is medium, and 0.1 is small [52].

## 3. Results

### 3.1. Descriptive Analyses

The Kolmogorov–Smirnov test indicated no significant deviation from normality for the Externalizing Problems subscale scores of the Child Behavior Checklist (*p* = 0.200) and for the Internalizing Problems (*p* = 0.156) and Total Problems (*p* = 0.200) subscale scores of the Youth Self-Report. In contrast, the Total Problems (*p* = 0.006) and Internalizing Problems (*p* = 0.021) subscales of the Child Behavior Checklist, along with the Externalizing Problems subscale of the Youth Self-Report (*p* = 0.037), demonstrated significant deviations from normality, necessitating the application of non-parametric analyses.

Clinical evaluation determined that over 90% of participants had at least one comorbidity, with 57.5% presenting two or more. In detail, clinical diagnoses, derived from structured interviews conducted in accordance with DSM-5 diagnostic criteria, identified in the sample included anxiety (68.5%), including generalized, social, and separation anxiety disorders, mood disorder (57.4%), including depression and bipolar disorder, ADHD (25.9%), Specific Learning Disorder (24.1%), Tic Disorders (9.3%), including Tourette’s Disorder, Behavioral Disorders (9.3%), including Oppositional Defiant, Disruptive, and Conduct Disorder, Obsessive-Compulsive Disorders (9.3%), and Feeding and Eating Disorder (3.7%), including anorexia, bulimia, and avoidant/restrictive food intake disorder.

In Table 1, we show descriptive analyses of the CBCL and YSR for each subscale.

**Figure 1:**
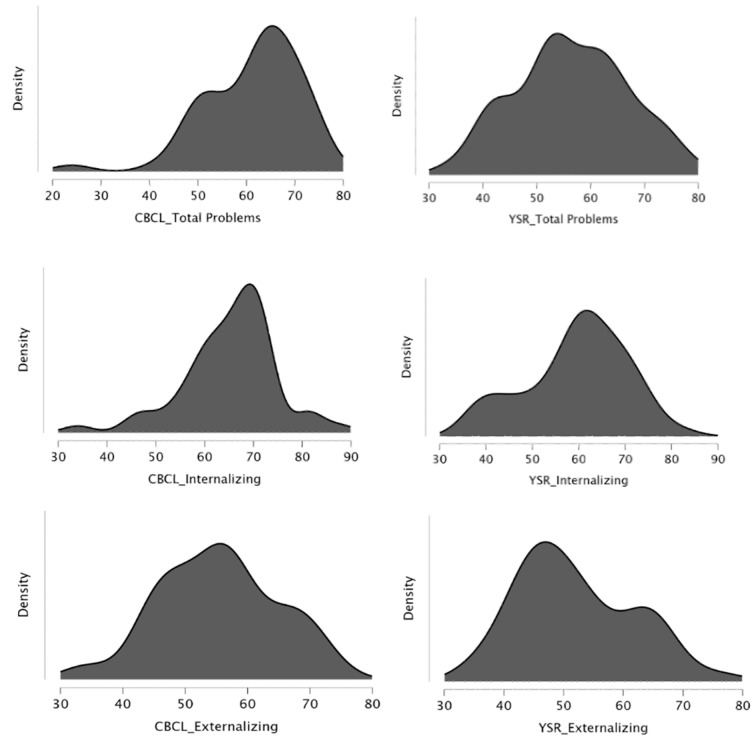
Frequency distribution of scores across the CBCL and YSR scales: Total Problems, Internalizing Problems, and Externalizing Problems scales.

The frequency distribution of T-scores for the CBCL summary scales revealed the following results (as shown in Figure 1):

− Total Problems scale: 16.7% of participants fell within the borderline range, while 50% were in the clinical range.
− Internalizing Problems scale: 16.7% of participants were in the borderline range, with 61.1% classified in the clinical range.
− Externalizing Problems scale: 7.4% of participants fell within the borderline range, and 20.4% were in the clinical range.

As shown in Figure 1, the frequency distribution of T-scores for the YSR summary scales demonstrated the following patterns:

− Total Problems scale: 18.5% of participants scored in the borderline range, and 24.1% fell within the clinical range.
− Internalizing Problems scale: 21.1% of participants were in the borderline range, while 33.3% were in the clinical range.
− Externalizing Problems scale: 7.4% of participants scored in the borderline range, and 18.5% fell within the clinical range.

### 3.2. Spearman’s Rank Correlation

The results of Spearman’s correlation showed no significant correlations between age and the variables of interest (all *p* > 0.05), suggesting that age did not have a meaningful influence on the variables under investigation.

### 3.3. Wilcoxon Signed-Rank Tests

**Figure 2:**
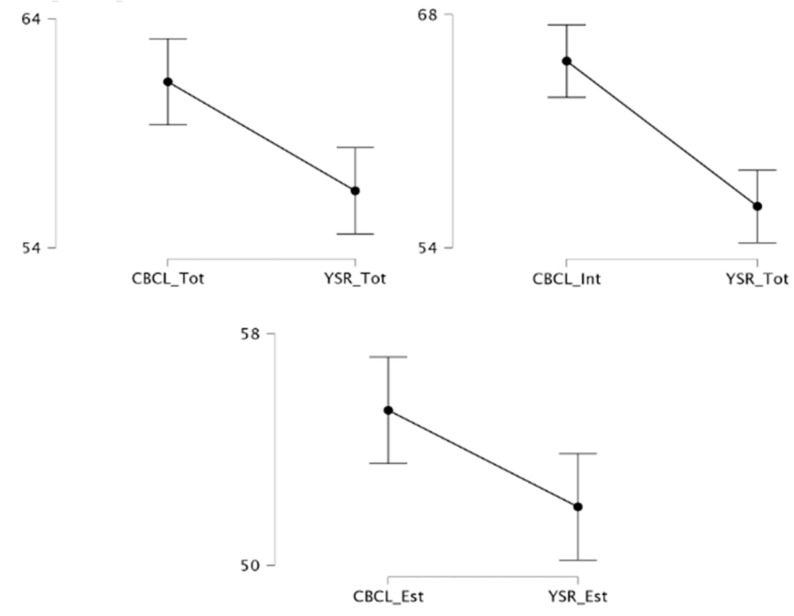
Paired-samples *t*-tests conducted to compare the CBCL and the YSR across the Total Problems (*p* < 0.001), Internalizing Problems (*p* < 0.001), and Externalizing Problems scales (*p* = 0.008).

The Wilcoxon Signed-Rank Test (Figure 2) conducted to compare scores obtained from the Child Behavior Checklist (CBCL) and the Youth Self-Report (YSR) across the Total Problems, Internalizing Problems, and Externalizing Problems scales show the following:

− Total Problems: the mean score for the CBCL Total Problems scale (M = 61.24, SD = 9.82) was significantly different from the YSR Total Problems scale (M = 56.48, SD = 10.40); W = 311.00, Z = −3.30, *p* < 0.001; with a medium effect size (r = −0.45);
− Internalizing Problems: the mean score for the CBCL Internalizing Problems scale (M = 65.20, SD = 9.48) was significantly different from the YSR Internalizing Problems scale (M = 59.56, SD = 10.80); W = 317.00, Z = −3.53, *p* < 0.001, with a medium effect size (r = −0.48).
− Externalizing Problems: the mean score for the CBCL Externalizing Problems scale (M = 55.35, SD = 9.31) was significantly different from the YSR Externalizing Problems scale (M = 52.02, SD = 9.66); W = 398.50, Z = −2.65, *p* = 0.008, with a medium effect size (r = −0.36).

## 4. Discussion

This study aimed to examine the level of agreement between parents and adolescents in the assessment of psychopathology among a sample of 54 autistic adolescents without intellectual impairment, using the CBCL/6-18 completed by parents and the YSR/11-18 completed by adolescents.

Among the autistic adolescents in our sample, 90% received a clinical diagnosis of at least one psychiatric co-occurring condition based on DSM-5 criteria. The prevalence of psychiatric conditions in this group was slightly higher than reported in previous studies [4,10,53]. Differences in prevalence estimates across studies may be attributed to various methodological factors, including classification systems, assessment tools, and demographic characteristics of the samples (e.g., mean age and sex distribution). Notably, in our study, co-occurring diagnoses were established through a comprehensive, multi-method assessment process based on DSM-5 criteria, rather than being solely derived from questionnaire scores or single-informant clinical interviews [54].

Regarding individual factors such as age, Mutler and colleagues (2022) conducted a meta-analysis of 39 studies examining the prevalence of psychiatric conditions in autistic children and adolescents [7]. Their findings indicate that autistic adolescents present with higher rates of co-occurring psychopathology compared to autistic children, aligning with previous meta-analyses and research on co-occurring conditions in autism.

Additionally, this study was conducted in a tertiary care hospital, where data were collected from autistic individuals with complex clinical presentations. This specialized setting may have contributed to the higher prevalence of co-occurring psychiatric conditions observed, compared to population-based studies [6].

In our sample, the most frequently diagnosed psychiatric conditions were anxiety disorders (68.5%), followed by mood disorders (57.4%) and ADHD (25.9%). These findings are consistent with the work of Lai and colleagues, who reported a higher prevalence of mood and anxiety disorders in older autistic individuals, whereas externalizing difficulties were more frequently observed in younger autistic children [4]. This is further supported by cross-sectional studies indicating that increasing age is a risk factor for the development of anxiety and mood disorders in both children [55] and adolescents [10].

The higher prevalence of anxiety and mood disorders observed in our sample, in line with the existing literature, suggests that in autistic adolescents without intellectual impairment, anxiety may be more closely linked to challenges in managing social interactions, adjusting to changes in daily routines, and regulating emotions. Furthermore, the high proportion of clinically diagnosed psychiatric conditions in our sample may be partially attributable to the emotional distress experienced by adolescents who receive a late autism diagnosis, particularly when their neurodivergence has not been recognized earlier in life. This highlights the need to develop clinical expertise specifically focused on assessing psychiatric conditions in autistic individuals and to validate useful assessment tools to distinguish between co-occurring conditions and manifestations that may be better understood as intrinsic to autism itself [56].

In line with our study objective, we examined how the description of psychiatric symptoms differs between parental reports and self-reports provided by autistic adolescents without intellectual impairment.

The CBCL and YSR are widely studied instruments used to assess emotional and behavioral difficulties in children and adolescents [16], including autistic individuals [40,55]. Emerging evidence supports the inclusion of the CBCL in assessment protocols for evaluating emotional and behavioral challenges in autistic children and adolescents, demonstrating good sensitivity but relatively low specificity in detecting these difficulties [51]. However, as highlighted by Shipkova and colleagues, relying solely on parental reports may be insufficient for obtaining a comprehensive understanding of internalizing and externalizing symptoms in autistic youth [56]. Clinicians and researchers emphasize the importance of incorporating multiple sources of information during diagnostic evaluations. In line with this, our study included both adolescent self-reports and parental reports [33].

Our findings align with previous research, as we observed a high prevalence of psychiatric co-occurring conditions diagnosed based on clinical judgment. These diagnoses were established through structured clinical interviews following DSM-5 diagnostic criteria and were further corroborated by the administration of the K-SADS-PL interview. Notably, the prevalence of these conditions appeared higher when determined through clinical evaluation than when reported by parents via the CBCL or by adolescents through the YSR.

In our sample, parents demonstrated greater awareness of their child’s emotional and behavioral difficulties than the adolescents themselves, though their reports still captured fewer concerns than clinical assessments.

Regarding the level of agreement between adolescent and parent reports using the ASEBA questionnaire, our results reveal significant discrepancies across the three summary scales, which assess emotional problems, behavioral difficulties, and their combined presentation—patterns frequently observed in autistic adolescents. These findings are consistent with previous studies on both typically developing adolescents and autistic youth [38].

Specifically, discrepancies were observed across the Internalizing, Externalizing, and Total Problems scales, with parents reporting more clinical concerns than adolescents. Significant differences between parent and adolescent reports have been documented in multiple studies [57,58]. In clinical samples, parents tend to report higher levels of symptoms than children and adolescents [40,59], whereas in typically developing samples, the opposite trend is often observed. The degree of agreement between parent and adolescent reports may be influenced by the social communication and emotional processing differences commonly associated with autism. These factors can impact an autistic adolescent’s ability to recognize and articulate their emotions, which in turn may limit their capacity for self-reporting and self-reflection [60,61,62]. Additionally, differences in introspective abilities and communication styles among autistic adolescents may contribute to difficulties in accurately identifying and expressing their mental health concerns, potentially delaying access to appropriate support and interventions.

However, not all studies align with this pattern of discordance. Empirical findings suggest that agreement levels between autistic adolescents and their parents may vary depending on the specific co-occurring condition being assessed. For example, greater agreement is often observed when reporting social difficulties and externalizing behaviors compared to internalizing symptoms such as anxiety or depression [40]. Furthermore, some studies using different assessment tools have found that autistic adolescents report higher levels of anxiety than both their typically developing peers and their own parents [20].

While our findings agree with a part of the literature, further research is needed to explore these discrepancies in greater depth. One limitation of this study is the relatively small sample size, particularly regarding gender differences in the presentation of co-occurring psychopathology. However, smaller cohorts can reduce variability and measurement errors, allowing for a more precise and detailed analysis, ultimately enhancing the reliability of the results [63]. Another limitation is that our study relied on clinical data collected at a tertiary university hospital in Italy. As the data were originally gathered for diagnostic and treatment purposes, we were unable to obtain additional contextual information about the participants (e.g., socio-demographic data), which may limit the generalizability of our findings to the broader autistic population. Future studies should aim to incorporate larger, more diverse samples and consider additional variables that may influence parent–adolescent agreement in the assessment of co-occurring mental health conditions.

Our study underscores the importance of integrating multiple sources of information in psychopathological assessments, as this approach provides a more comprehensive understanding of symptoms, reduces the risk of misinterpretation or symptom underreporting, and ultimately facilitates more targeted and effective interventions. By incorporating perspectives from different informants, the characterization of co-occurring conditions in autism becomes more precise, fostering a deeper understanding of these challenges and promoting clearer communication between adolescents and their parents.

A key strength of this study is the inclusion of an Italian sample of autistic adolescents without intellectual disabilities or language difficulties, whose diagnoses were established through clinical assessments based on DSM-5 criteria, conducted by expert clinicians using gold-standard diagnostic tools. We highlight the importance of employing the CBCL within a multi-method, multi-informant framework to support clinical judgment, aid in differential diagnosis, and improve the identification of co-occurring mental health conditions in autistic adolescents without intellectual impairment.

A major limitation in the field remains the lack of psychopathological assessment tools specifically designed for autistic individuals that incorporate both self-report and parent-report versions. This limitation necessitated the use of an instrument that, while offering both perspectives, was not explicitly developed for the autistic population. Therefore, it is essential to refine and develop assessment tools tailored to the specific cognitive, emotional, and communication profiles of autistic individuals, which could enhance the reliability of inter-informant agreement analyses and improve diagnostic accuracy.

Future research should explore protocols in which additional informants, such as teachers, clinicians, or peers, contribute to the assessment process. This would allow for a more comprehensive understanding of symptom presentation across different social and environmental contexts. Moreover, studies should aim to include larger and more diverse samples, with balanced gender representation and a broader age range, to improve the generalizability of findings. The development of autism-specific assessment tools will also be critical in advancing research and clinical practice in this area.

## 5. Conclusions

Overall, the findings of this study emphasize the importance of integrating multiple informants to ensure a more accurate and comprehensive assessment of psychiatric conditions associated with autism. This approach promotes a holistic understanding of an individual’s behavioral and emotional experiences across different life contexts, ultimately enhancing clinical evaluation and support strategies.

## Figures and Tables

**Table 1:** Descriptive analyses of CBCL and YSR subscales.

	Mean	Std. Deviation	Skewness	Std. Error of Skewness	Kurtosis	Std. Error of Kurtosis	Minimum	Maximum
CBCL_Internalizing Problems	65.20	9.48	−0.65	0.32	1.77	0.64	34.00	88.00
CBCL_Externalizing Problems	55.35	9.31	−0.04	0.32	−0.37	0.64	34.00	74.00
CBCL_Total Problems	61.24	9.82	−1.16	0.32	2.44	0.64	24.00	76.00
CBCL_Affective Problems	64.89	9.19	0.22	0.32	−0.64	0.64	50.00	85.00
CBCL_Anxiety Problems	65.57	7.85	−0.28	0.32	−0.16	0.64	50.00	85.00
CBCL_Somatic Problems	56.53	7.52	0.95	0.33	−0.01	0.64	50.00	77.00
CBCL_Attention Deficit/Hyperactivity Problems	58.61	8.50	1.23	0.32	2.03	0.64	50.00	90.00
CBCL_Oppositional Defiant Problems	57.56	6.73	0.80	0.32	−0.46	0.64	50.00	75.00
CBCL_Conduct Problems	54.39	5.74	1.08	0.32	−0.46	0.64	50.00	67.00
CBCL_Anxiety/Depressed	63.93	8.29	0.01	0.32	−0.35	0.64	50.00	85.00
CBCL_Withdrawn/Depressed	68.80	11.95	0.40	0.32	−0.52	0.64	50.00	93.00
CBCL_Somatic Complains	59.11	8.68	1.09	0.32	1.30	0.64	50.00	89.00
CBCL_Social Problems	63.52	7.63	−0.21	0.32	−0.44	0.64	50.00	80.00
CBCL_Thought Problems	62.06	9.38	0.06	0.32	−1.47	0.64	50.00	79.00
CBCL_Attention Problems	61.28	9.13	0.96	0.32	1.44	0.64	50.00	93.00
CBCL_Rule Breaking Behavior	54.87	5.13	1.02	0.32	−0.26	0.64	50.00	67.00
CBCL_Aggressive Behavior	57.85	7.94	0.84	0.32	−0.31	0.64	50.00	79.00
YSR_Internalizng Problems	59.56	10.80	−0.45	0.32	−0.34	0.64	36.00	82.00
YSR_Externalizing Problems	52.02	9.66	0.48	0.32	−0.44	0.64	34.00	77.00
YSR_Total Problems	56.48	10.40	0.02	0.32	−0.57	0.64	34.00	79.00
YSR_Affective Problems	59.06	8.34	0.49	0.32	−1.00	0.64	50.00	78.00
YSR_Anxiety Problems	60.91	8.44	−0.01	0.32	−1.37	0.64	50.00	78.00
YSR_Somatic Problems	56.19	7.75	1.50	0.32	1.68	0.64	50.00	81.00
YSR_CBCL_Attention Deficit/Hyperactivity Problems	56.13	5.94	0.81	0.32	−0.65	0.64	50.00	70.00
YSR_CBCL_Oppositional Defiant Problems	56.30	6.32	1.03	0.32	−0.03	0.64	50.00	73.00
YSR_Conduct Problems	54.35	6.67	1.92	0.32	3.42	0.64	50.00	78.00
YSR_Anxiety/Depressed	61.52	9.14	0.68	0.32	0.10	0.64	50.00	85.00
YSR_Withdrawn/Depressed	61.70	10.89	0.83	0.32	−0.40	0.64	50.00	87.00
YSR_Somatic Complains	57.00	7.97	1.36	0.32	1.37	0.64	50.00	80.00
YSR_Social Problems	60.02	8.76	1.71	0.32	4.53	0.64	50.00	95.00
YSR_Thought Problems	58.13	9.91	1.48	0.32	1.75	0.64	50.00	91.00
YSR_Attention Problems	56.56	6.93	1.12	0.32	0.65	0.64	50.00	77.00
YSR_Rule Breaking Behavior	52.94	5.46	2.64	0.32	7.76	0.64	50.00	77.00
YSR_Aggressive Behavior	56.57	7.60	1.06	0.32	−0.07	0.64	50.00	77.00

CBCL (Child Behavior Checklist), YSR (Youth Self-Report).

## Data Availability

The raw data supporting the conclusions of this article will be made available by the authors upon reasonable request. The data are not publicly available because of privacy concerns.
